# *Clostridium difficile* in western Romania: unfavourable outcome predictors in a hospital for infectious diseases

**DOI:** 10.1186/s12879-015-0895-y

**Published:** 2015-03-21

**Authors:** Ruxandra Laza, Ruxandra Jurac, Alexandru Crişan, Voichiţa Lăzureanu, Monica Licker, Emilian Damian Popovici, Luminiţa Mirela Bădiţoiu

**Affiliations:** Department 13, Infectious Diseases II Department, “Victor Babeş” University of Medicine and Pharmacy, Timişoara, Romania; “Dr. Victor Babeş” Infectious Diseases and Pneumophtisiology Hospital, Timişoara, Romania; “Louis Ţurcanu” Emergency Hospital for Children, Timişoara, Romania; Department 14, Microbiology Department, “Victor Babeş” University of Medicine and Pharmacy, Timişoara, Romania; Department 13, Epidemiology Department, “Victor Babeş” University of Medicine and Pharmacy, Timişoara, Romania; Regional Public Health Center Timişoara, 16 Victor Babeş, Timişoara, Romania

**Keywords:** *Clostridium difficile*, Nosocomial infections, Emerging pathology

## Abstract

**Background:**

The recent emergence of *Clostridium difficile* infections has included this condition among top nosocomial infections, due to its incidence, complications and important fatality, as well as to significant economic costs.

**Methods:**

A prospective surveillance study of *Clostridium difficile* enterocolitis cases was performed in “Victor Babeş” Infectious Diseases Hospital in Timişoara (Romania) between 01.01.2013 - 30.06.2014, to estimate the incidence and to investigate the risk factors for unfavourable outcome and relapse. Dichotomous variables were compared by the chi-square test or Fisher exact test and the Mann–Whitney U test was used for continuous variables. Risk factors for unfavourable outcome/recurrence were investigated by logistic regression.

**Results:**

210 patients who experienced 219 episodes of infection with *Clostridium difficile* were identified, which gives an incidence per hospital of 20.57/15.70 to 1,000 discharged patients in 2013/2014 or 17.73/14.04 to 10,000 patient-days. In 162 patients (77.14%) the evolution was favourable while in 48 (22.86%) the outcome was unfavourable. In 42 patients (20.00%) recurrence of symptoms was identified. The multivariate analysis by logistic regression identified the ATLAS score (OR = 4.97, 95% CI = 2.12 to 11.66, p <0.001), age (OR = 1.12, 95% CI = 1.00 to 1.25, p = 0.046), and the number of antibiotics after episode onset (OR = 2.692, 95% CI = 1.01 to 7.17, p = 0.047) as predictors of an unfavourable evolution, while the number of hospitalization days (OR = 1.10, 95% CI = 1.03 to 1.16, p = 0.0015) was associated with recurrence of symptoms.

**Conclusions:**

The high incidence identified in our study is explained by the endemic character of these infections in some hospitals in Timişoara, released in late 2012, and the fact that “Victor Babeş” Hospital is the only one in our area that provides treatment in all suspected or confirmed cases of this condition requiring hospitalization. The study identified the ATLAS score, age, and the number of antibiotics after episode onset as predictors of unfavourable evolution, while the number of days of hospitalization was associated with the recurrence of symptoms.

## Background

The emergence of *Clostridium difficile*-associated disease (CDAD) in recent years has imposed this condition among nosocomial infections, by incidence, significant complications, and fatality, as well as significant economic costs.

Surveillance results on the point prevalence of nosocomial infections and their relation of causality with the use of antibiotics in European hospitals, in studies conducted by the European Centre for Disease Prevention and Control showed that 5.4% healthcare-associated infections with documented bacterial aetiology were determined by *Clostridium difficile* (CD) [1].

According to a 2013 study on the point prevalence of healthcare associated infections in long-term care institutions in Europe, CD was identified as causative agent of gastrointestinal infections in 12.1% of cases in Italy, 18.2% in Ireland and 20.6% in Germany [2].

In Barcelona hospitals, in 2009, the incidence varied between 0 and 5.97 to 1,000 discharged patients and, according to a study from 2011, the annual incidence in Spain is estimated at 1.71 cases per 1,000 admitted patients [3,4].

An evaluation performed by the European Centre for Disease Prevention and Control - European Society of Clinical Microbiology and Infectious Diseases estimates the direct cost of care in Europe at 3,700 million euros (updated data for 2013) and 7,000-7,500 euros/hospitalized patient in Italy, Germany and Britain [5]. In Romania, the increase of the CDAD incidence started in 2011, when these cases started to be investigated for CD by appropriate bacteriological tests, and in 2013, 1,237 cases were reported nationwide, with a significantly higher proportion of the 027 ribotype, a strain with increased transmissibility rates/sporogenesis, increased production of A and B specific toxins and additional binary toxin production [5].

The interest aroused by the frequency of this pathology (not identified in our region until 2012), is all the more justified since, after its identification by Holl and O'Tooll in 1935, for 43 years, this bacterium was only labelled as saprophytic, included in the normal resident flora of the digestive tract. Clinical observations after 1978, but especially in recent years, bring evidence of certain changes in the clinical course and epidemiology of CD infections. An increase in the number of severe forms of illness is recorded, more frequently affecting patients over 65 years, together with higher numbers of community acquired cases and of individuals previously considered at minor risk who develop the disease.

In this context, the aims of our study were to estimate the incidence and to clarify some epidemiological aspects regarding the CDAD in an Infectious Diseases Hospital providing healthcare for the population of Timis county (located in western Romania) and to identify the predictive factors for unfavourable outcome.

## Methods

### Study design

We conducted a prospective surveillance study, including all patients diagnosed with CDAD, hospitalized between 01.01.2013 - 30.06.2014 at the Clinical Hospital of Infectious Diseases and Pneumophtisiology “Victor Babeş” Timişoara, an hospital with 120 beds, with 7,194 discharged patients in 2013/3,949 in 2014 and 83,484 patient-days in 2013/44,146 in 2014.

We only included laboratory confirmed cases in patients aged 18 or older, regardless of gender, personal history and probable infection source i.e. nosocomial or community acquired. After being discharged, the patients were surveyed for three months in order to detect possible recurrence and unfavourable evolutions. The database included only the first episode of CDAD for each patient.

We collected demographic data, the total number of days of hospitalization for CDAD, the presence of various comorbidities (malignancy, diabetes, chronic renal failure, cardiac, pulmonary, mild/moderate/severe liver pathology, or peripheral vascular, cerebrovascular, haematological diseases, dementia, gastro-duodenal ulcer, presence of concomitant infections); possible causes of immunosuppression during the last 2 months prior to the onset of CDAD (chemotherapy, radiotherapy, corticosteroids, chronic dialysis, surgery - including the type of intervention); other risk factors (inflammatory bowel disease, colorectal cancer, previous exposure to antimicrobial agents, enteral/parenteral nutrition), and the possible unfavourable evolution (continuing the administration of drugs which slow down intestinal motility, proton pump inhibitors, other antacids, antibiotics - other than those for CD, specifying the number of days and number of antibiotics received); clinical data (serum albumin, blood leukocyte count, body temperature) with the calculation of the ATLAS Score, recording progress and treatment received.

The case definition for CDAD included the presence of the diarrheal syndrome (≥3 stools per day) or toxic megacolon. The aetiology was confirmed by the VIDAS® *C. difficile* Toxin A & B (bioMérieux) test, an ELFA (Enzyme-Linked Fluorescent Assay) that detects toxins A and B in fresh stool samples. In patients with positive symptoms but negative tests for toxins, the test was repeated after 48-72 h.

The “Unfavourable outcome” was defined as death within 30 days after onset of the episode (in hospital/at home) or complications requiring transfer to a surgical ward. The recurrence of symptoms within less than 8 weeks was classified as relapse and after this limit as recidivation.

The study was approved by the Ethics Committee of the “Victor Babeş” Hospital for Infectious Diseases and Pneumophtisiology Timişoara and the informed consent was obtained from all patients included. Throughout the study, the patients received standard treatments and were not subjected to any further clinical intervention or investigation.

### Statistical analysis

The database was processed using the SPSS software version 10.0. Continuous numeric variables were characterized by median and range of quartile (IQR) and the category type by value and percentage. Testing data distribution was performed using the Kolmogorov-Smirnov test. Comparison of dichotomous variables was performed by chi-square test or Fisher exact test and the Mann–Whitney U test was used for continuous variables. Statistical significance was calculated by two-tailed tests and significance threshold was set at p values ≤ 0.05. The correlation of the ATLAS Score with the Charlson comorbidity index was obtained using the Spearman coefficient. Risk factors for unfavourable outcome/recurrence were investigated by logistic regression. Independent variables with p ≤0.10 in the univariate analysis were entered into multivariate sequenced analysis to obtain Odds ratio and 95% confidence interval. To avoid colinearity, 2 options were used: i - with the inclusion of the multifaceted independent variables, but without their constituent variables; ii - including only simple independent variables. Model selection was performed depending on the Nagelkerke R^2^ coefficient and the deviation from the theoretical model, estimated by the Hosmer and Lemeshow Goodness of fit test.

## Results

210 patients who experienced 219 episodes of CDAD were identified, yielding an incidence per hospital of 20.57/15.70 to 1,000 discharged patients in 2013/2014 (95% CI 17.2-24.1/12.2-20.3) or 17.73/14.04 to 10,000 patient-days (95% CI 10.2-30.1/10.3-20.2) (p = 0.070/0.122). The demographic characteristics and co-morbidities in the study group (N = 210) are shown in Table 1.

**Table 1:** **Demographic characteristics and comorbidity of the sample included in the study (N = 210 cases)**

**The characteristic**	**Identified value**
**Demographic characteristics**	
Median age [years (IQR)]	68 (57–77)
Female gender [n (%)]	110 (52.38)
Urban area [n (%)]	146 (69.52)
County of residence Timiş [n (%)]	181 (86.20)
County of residence Caraş-Severin [n (%)]	10 (4.76)
County of residence Hunedoara [n (%)]	5 (2.38)
County of residence Arad [n (%)]	1 (0.47)
Other counties [n (%)]	13 (6.19)
**Clinical and evolutive features**	
Nosocomial infection with CD [n (%)]	204 (97.14)
Community infection with CD [n (%)]	6 (2.86)
Patients cured [n (%)]	162 (77.14)
Patients deceased in the hospital [n (%)]	40 (19.05)
Patients deceased later, at home [n (%)]	5 (2.38)
Transferred patients [n (%)]	3 (1.43)
Relapses [n (%)]	42 (20)
Median of the no.of days of hospitalization for CDAD [days (IQR)]	10 (7–13)
Median no. of days between the first CDAD episode and the recurrence of symptoms [days (IQR)]*	14 (7–21)
Median no. of leucocytes [cells/μl (IQR)]	10040 (7210–14800)
Leukocytosis ≥ 16.000 cells/μl [n (%)]	44 (20.95)
Hypoalbuminemia <3,5 g/dL [n (%)]	166 (79.05)
Fever > 37.5°C [n (%)]	51 (24.49)
Median ATLAS score (IQR)	3 (2–4)
Mild clinical form [n (%)]	7 (3.33)
Moderate clinical form [n (%)]	153 (72.86)
Sever clinical form [n (%)]	50 (23.81)
**Comorbidities**	
Median Charlson comorbidity index score (IQR)	2.5 (1–4)
Median Charlson comorbidity Index score adjusted for age (IQR)	5 (1–5)
Malignancy [n (%)]	44 (20.95)
Colorectal cancer [n (%)]	5 (2.38)
Diabetes mellitus [n (%)]	48 (22.86)
Renal disease [n (%)]	47 (22.38)
Chronic renal failure [n (%)]	35 (16.67)
Chronic heart disease [n (%)]	111 (52.86)
Peripheral vascular disease [n (%)]	40 (19.05)
Dementia [n (%)]	10 (4.76)
Cerebrovascular disease [n (%)]	55 (26.19)
Chronic pulmonary pathology [n (%)]	38 (18.09)
Moderate/severe liver pathology [n (%)]	26 (12.38)
Ulcerous disease [n (%)]	23 (10.95)
Inflammatory bowel disease [n (%)]	5 (2.38)
Haematological pathology [n (%)]	6 (2.86)
Concomitant infections [n (%)]	59 (28.09)
**Risk factors for CDAD in the past 2 months**	
Chemotherapy [n (%)]	14 (6.67)
Radiotherapy [n (%)]	7 (3.33)
Corticotherapy [n (%)]	15 (7.14)
Dialysis [n (%)]	7 (3.33)
Surgery [n (%)]	97 (46.19)
Gastro-intestinal/abdominal surgery [n (%)]	43 (20.48)
Enteral feeding [n (%)]	4 (1.90)
Parenteral feeding [n (%)]	22 (10.48)
Prior antibiotic treatment [n (%)]	145 (69.05)
Intestinal motility inhibitors after CDAD onset [n (%)]	20 (9.52)
Proton pump inhibitors after CDAD onset [n (%)]	22 (10.48)
Other antacids after CDAD onset [n (%)]	65 (30.95)
Antibiotic use after CDAD diagnosis (other than for CDAD) [n (%)]	32 (15.24)
Median no. of days of antibiotherapy after onset of episode (other than the treatment for CDAD) [days (IQR)]	0 (0–0)
Median no. of antibiotics after onset of episode (other than the treatment for CDAD [no. (IQR)]	0 (0–0)
**Treatment for CDAD**	
Metronidazole [n (%)]	42 (20)
Vancomycin [n (%)]	58 (27.62)
Vancomycin + Metronidazole [n (%)]	99 (47.14)
Rifaximin - α (alone or in combination) [n (%)]	9 (4.28)
Death before initiating the therapy [n (%)]	2 (0.95)

*Calculated for the subsample of relapse (N = 42).

**Figure 1:**
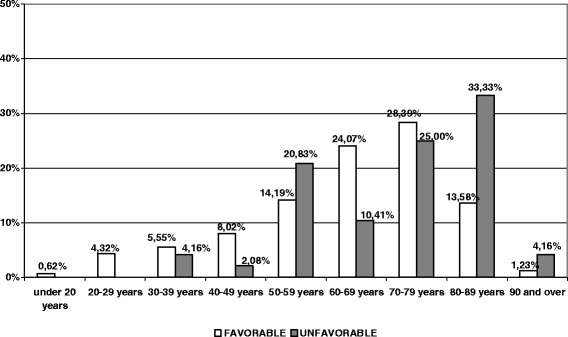
**Distribution of cases by age group and CDAD development.**

The most affected were patients aged between 70–79 years, followed by those aged 60–69 and 80–89, as shown in Figure 1.

162 patients (77.14%) responded favourably, while 48 (22.86%) had an unfavourable outcome. 145 (69.05%) had received prior antibiotic therapy, most of them received IIIrd generation cephalosporins (31.03%) and fluoroquinolones (28.96%), followed by carbapenems (17.93%) and β-lactam with β-lactamase inhibitor (15.86%). Two patients (0.95%) died before the introduction of therapy, but the remaining 208 were treated, 99 (47.14%) with Metronidazole + Vancomycin, 58 (27.62%) with Vancomycin and 42 (20%) with Metronidazole.

The ATLAS Score - known for its ability to predict the unfavourable outcome correlated directly, statistically significant, with the Charlson comorbidity index (weak correlation) (r = 0.356, p <0.001) and with the age-adjusted comorbidity index (medium correlation) (r = 0.505, p <0.001). Also, a medium correlation was detected between the ATLAS score and the evolution of cases (r = 0.57, p <0.001).

The univariate analysis identified several risk factors for the unfavourable outcome: age, leukocytosis over 16,000 cells/μl, hypoalbuminemia <3.5 g/dL, fever over 37.5°C, chronic lung pathology and moderate/severe liver pathology, chemotherapy, parenteral nutrition, antibiotic therapy continued after the onset of the episode (other than therapy for CDAD), number of days and number of antibacterial agents (other than CDAD therapy), the ATLAS score and the Charlson comorbidity index, simple or age adjusted. (Table 2) Of these, multivariate analysis retained the ATLAS score (OR = 4.97, 95% CI = 2.12 to 11.66, p <0.001) in the first model, while in option ii age it retained (OR = 1.12, 95% CI = 1.00 to 1.25, p = 0.046) and the number of antibiotics after episode onset, other than CDAD therapy (OR = 2.692, 95% CI = 1.01 to 7.17, p = 0.047).

**Table 2:** **Risk factors for unfavourable outcome of CDAD**

**Variable**	**Favorable**	**Unfavourable**	**Univariate analysis**		**Multivariate analysis**	
	**(N = 162)**	**(N = 48)**	**p**	**OR (95% CI)**	**p**	**OR (95% CI)**
**Demographic characteristics**						
Median age [years (IQR)]	67 (57–75)	76 (58–81)	0.007	1.03 (1.00-1.05)	0.046	1.12 (1.00-1.25)
Female gender [n (%)]	86 (53.09)	24 (50)	0.707	0.88 (0.44-1.77)		
Urban area [n (%)]	118 (72.84)	28 (58.33)	0,055	1.92 (0.93-3.94)		
**Clinical and evolutive characteristics**						
Nosocomial infection with CD [n (%)]	156 (96.30)	48 (100)	0.340	0.00 (0–2.87)		
Relapses [n (%)]	30 (18.52)	12 (25)	0.324	1.47 (0.64-3.35)		
Median no. of hospitalization days for CDAD [days (IQR)]	10 (8–13)	4 (2.5-12.5)	0.002	0.88 (0.82-0.95)		
Median no. of leukocytes [cells/μl (IQR)]	9565 (7210–32400)	16100 (7120–20135)	<0.001	1.00 (1.0001-1.0002)		
Leukocytosis ≥ 16000 cells/μl [n (%)]	19 (11.73)	25 (52.08)	<0.001	8.18 (3.67-18.43)		
Hypoalbuminemia < 3.5 g/dl [n (%)]	119 (73.46)	47 (97.92)	<0.001	16.98 (2.70-700.55)		
Fever > 37.5°C [n (%)]	21 (12.96)	31 (64.58)	<0.001	12.24 (5.45-27.88)		
Median ATLAS Score (IQR)	2 (1–3)	5 (4–6)	<0.001	2.72 (2.02-3.67)	<0.001	4.97 (2.12-11.66)
Mild clinical form [n (%)]	7 (4.32)	0 (0)	0.355	0.00 (0.00-2.33)		
Moderate clinical form [n (%)]	150 (92.59)	3 (6.25)	<0.001	0.01 (0.00-0.02)		
Sever clinical form [n (%)]	5 (3.09)	45 (93.75)	<0.001	471 (94.88-2836.91)		
**Comorbidities**						
Median Charlson comorbidity index score (IQR)	2 (1–3)	4 (3–5.5)	<0.001	1.48 (1.26-1.73)		
Median Charlson comorbidity index score adjusted for age (IQR)	4.5 (2–6)	7 (5.5-8.5)	<0.001	1.40 (1.22-1.60)		
Malignancy [n (%)]	31 (19.14)	13 (27.08)	0.234	1.57 (0.69-3.52)		
Colorectal cancer [n (%)]	5 (3.09)	0 (0)	0.590	0.00 (0.00-3.70)		
Diabetes mellitus [n (%)]	35 (21.61)	13 (27.08)	0.427	1.35 (0.60-2.99)		
Renal disease [n (%)]	35 (21.61)	12 (25)	0.620	1.21 (0.53-2.72)		
Chronic renal failure [n (%)]	23 (14.20)	12 (25)	0.078	2.01 (0.85-4.73)		
Chronic heart disease [n (%)]	80 (49.38)	31 (64.58)	0.064	1.87 (0.91-3.85)		
Peripheral vascular disease [n (%)]	28 (17.28)	12 (25)	0.231	1.60 (0.69-3.66)		
Dementia [n (%)]	7 (4.32)	3 (6.25)	0.699	1.48 (0.24-6.78)		
Cerebrovascular disease [n (%)]	37 (22.84)	18 (37.50)	0.042	2.03 (0.96-4.27)		
Chronic pulmonary pathology [n (%)]	23 (14.20)	15 (31.25)	0.007	2.75 (1.21-6.22)		
Moderate/severe liver pathology [n (%)]	15 (9.26)	11 (22.92)	0.011	2.91 (1.14-7.43)		
Gastrointestinal ulcer [n (%)]	16 (9.88)	7 (14.58)	0.359	1.56 (0.54-4.38)		
Inflammatory bowel disease [n (%)]	4 (2.47)	1 (2.08)	1	0.84 (0.02-8.77)		
Haematological pathology [n (%)]	2 (1.23)	4 (8.33)	0.025	7.27 (0.99-81.83)		
Concomitant infections [n (%)]	44 (27.16)	16 (33.33)	0.405	1.34 (0.63-2.83)		
**Risk factors for CDAD in the past 2 months**						
Chemotherapy [n (%)]	7 (4.32)	7 (14.58)	0.020	3.78 (1.11-12.87)		
Radiotherapy [n (%)]	5 (3.09)	2 (4.17)	0.660	1.37 (0.13-8.67)		
Corticotherapy [n (%)]	9 (5.56)	6 (12.5)	0.115	2.43 (0.67-8.11)		
Dialysis [n (%)]	6 (3.70)	1 (2.08)	1	0.55 (0.01-4.75)		
Surgery [n (%)]	82 (50.62)	15 (31.25)	0.018	0.44 (0.21-0.92)		
Gastro-intestinal/abdominal surgery [n (%)]	37 (22.84)	6 (12.5)	0.118	0.48 (0.17-1.30)		
Enteral feeding [n (%)]	1 (0.62)	3 (6.25)	0.038	10.73 (0.83-566.82)		
Parenteral feeding [n (%)]	5 (3.09)	17 (35.42)	<0.001	17.22 (5.47-62.96)		
Prior antibiotic treatment [n (%)]	115 (70.99)	30 (62.50)	0.264	1.47 (0.71-3.04)		
Intestinal motility inhibitors after CDAD onset	17 (10.49)	3 (6.25)	0.576	0.57 (0.10-2.10)		
Proton pump inhibitors after CDAD onset [n (%)]	16 (9.88)	6 (12.5)	0.602	1.30 (0.39-3.78)		
Other antacids after CDAD onset [n (%)]	45 (27.78)	20 (41.67)	0.067	1.86 (0.90-3.82)		
Antibiotic use after CDAD diagnosis (other than for CDAD) [n (%)]	15 (9.26)	17 (35.42)	<0.001	5.37 (2.26-12.84)		
Median no. days of antibiotherapy after onset of episode (other than the treatment for CDAD)	0 (0–0)	0 (0–3)	0.017	1.09 (1.01-1.18)		
Median no. antibiotics after onset of episode (other than the treatment for CDAD) [no. (IQR)]	0 (0–0)	0 (0–1)	0.002	1.74 (1.21-2.50)	0.047	2.69 (1.01-7.17)
**Treatment for CDAD**						
Metronidazole [n (%)]	36 (22.22)	6 (12.5)	0.139	0.50 (0.16-1.32)		
Vancomycin [n (%)]	47 (29.01)	11 (22.92)	0.406	0.73 (0.32-1.63)		
Vancomycin + Metronidazole [n (%)]	71 (43.83)	28 (58.33)	0.077	1.79 (0.89-3.63)		
Rifaximin - α (alone or in combination) [n (%)]	8 (4.93)	1 (2.08)	0.687	0.41(0.01-3.20)		

A second comparison aimed at the identification of the risk factors for recurrence of symptoms induced by CD. In our study, 42 patients (20.00%) were identified with recurrence. While the univariate analysis associated the number of days of hospitalization, leukocytosis over 16,000 cells/μl, hypoalbuminemia <3.5 g/dL, the ATLAS Score and severe clinical form of recurrence risk, after the multivariate logistic regression model, this association remained only in terms of the number of days of hospitalization (OR = 1.10, 95% CI = 1.03 to 1.16, p = 0.0015) (Table 3).

**Table 3:** **Risk factors for CDAD relapse**

**Variable**	**Single episode**	**Recurrence of symptoms**	**Univariate analysis**		**Multivariate analysis**	
	**(N = 168)**	**(N = 42)**	**p**	**OR (95% CI)**	**p**	**OR (95% CI)**
**Demographic characteristics**						
Median age [years (IQR)]	67.5 (57–76)	74 (58–82)	0.266	1.01 (0.99-1.03)		
Female gender [n (%)]	86 (51.19)	24 (57.14)	0.489	1.27 (0.61-2.66)		
Urban area [n (%)]	111 (66.07)	35 (83.33)	0.029	0.39 (0.14-0.97)		
**Clinical and evolutive characteristics**						
Nosocomial infection with CD [n (%)]	164 (97.62)	40 (95.24)	0.344	2.05 (0.18-14.83)		
Median hospitalization for CDAD [days (IQR)]	10 (7–13)	12 (8–17)	0.004	1.12 (1.05-1.19)	0.0015	1.10 (1.03-1.16)
Median no. of leukocytes [cells/μl (IQR)]	9615 (6930–13820)	12625 (7830–17230)	0.277	1.00 (1.00-1.001)		
Leukocytosis ≥ 16000 cells/μl [n (%)]	29 (17.26)	15 (35.71)	0.008	2.66 (1.18-5.99)		
Hypoalbuminemia <3.5 g/dl [n (%)]	127 (75.59)	39 (92.86)	0.014	4.2 (1.22-22.22)		
Fever > 37.5°C [n (%)]	40 (23.81)	13 (30.95)	0.340	1.43 (0.64-3.20)		
Median ATLAS Score (IQR)	2 (2–4)	4 (2–5)	0.004	1.27 (1.07-1.51)		
Mild clinical form [n (%)]	7 (4.17)	0 (0)	0.349	0.00 (0.00-2.78)		
Moderate clinical form [n (%)]	127 (75.59)	26 (61.90)	0.074	0.52 (0.24-1.14)		
Severe clinical form [n (%)]	34 (20.24)	16 (38.09)	0.015	2.43 (1.10-5.33)		
**Comorbidities**						
Median Charlson comorbidity index score (IQR)	2.5 (1–4)	2.5 (1–4)	0.775	1.02 (0.88-1.18)		
Median Charlson comorbidity Index score adjusted for age (IQR)	5 (3–7)	5 (4–7)	0.446	1.04 (0.93-1.16)		
Malignancy [n (%)]	34 (20.24)	10 (23.81)	0.611	1.23 (0.51-2.93)		
Colorectal cancer [n (%)]	5 (2.98)	0 (0)	0.585	0.00 (0.00-4.40)		
Diabetes mellitus [n (%)]	39 (23.21)	9 (21.43)	0.805	0.90(0.37-2.189		
Renal disease [n (%)]	40 (23.81)	7 (16.67)	0.320	0.64 (0.24-1.65)		
Chronic renal failure [n (%)]	29 (17.26)	6 (14.29)	0.643	0.8 (0.25-2.16)		
Chronic heart disease [n (%)]	85 (50.59)	26 (61.90)	0.189	1.59 (0.75-3.36)		
Peripheral vascular disease [n (%)]	30 (17.86)	10 (23.81)	0.379	1.44 (0.59-3.46)		
Dementia [n (%)]	7 (4.17)	3 (7.14)	0.422	1.77 (0.28-8.17)		
Cerebrovascular disease [n (%)]	42 (25)	13 (30.95)	0.432	1.34 (0.60-2.99)		
Chronic pulmonary pathology [n (%)]	30 (17.86)	8 (19.05)	0.857	1.08 (0.41-2.75)		
Moderate/severe liver pathology [n (%)]	19 (11.31)	7 (16.67)	0.345	1.57 (0.55-4.35)		
Gastrointestinal ulcer [n (%)]	18 (10.71)	5 (11.90)	0.786	1.13 (0.31-3.42)		
Inflammatory bowel disease [n (%)]	4 (2.38)	1 (2.38)	1	1 (0.02-10.46)		
Haematological pathology [n (%)]	5 (2.98)	1 (2.38)	1	0.80 (0.02-7.40)		
Concomitant infections [n (%)]	48 (28.57)	12 (28.57)	1	1 (0.44-2.24)		
**Risk factors for CDAD in the past 2 months**						
Chemotherapy [n (%)]	12 (7.14)	2 (4.76)	0.740	0.65 (0.07-3.11)		
Radiotherapy [n (%)]	5 (2.98)	2 (4.76)	0.629	1.63 (0.15-10.38)		
Corticotherapy [n (%)]	14 (8.33)	1 (2.38)	0.313	0.27 (0.01-1.87)		
Dialysis [n (%)]	6 (3.57)	1 (2.38)	1	0.66 (0.01-5.67)		
Surgery [n (%)]	78 (46.43)	19 (45.24)	0.889	0.95 (0.46-1.98)		
Gastro-intestinal/abdominal surgery [n (%)]	36 (21.43)	7 (16.67)	0.493	0.73 (0.27-1.91)		
Enteral feeding [n (%)]	4 (2.38)	0 (0)	0.586	0.00 (0.00-6.12)		
Parenteral feeding [n (%)]	19 (11.31)	3 (7.14)	0.578	0.60 (0.11-2.21)		
Prior antibiotic treatment [n (%)]	120 (71.43)	25 (59.52)	0.135	1.70 (0.80-3.62)		
Intestinal motility inhibitors after CDAD onset	15 (8.93)	5 (11.90)	0.560	1.38 (0.37-4.32)		
Proton pump inhibitors after CDAD onset [n (%)]	21 (12.50)	1 (2.38)	0.086	0.17 (0.00-1.13)		
Other antacids after CDAD onset [n (%)]	54 (32.14)	11 (26.19)	0.455	0.75 (0.33-1.69)		
Antibiotics after CDAD diagnosis (other than the treatment for CDAD) [n (%)]	23 (13.69)	9 (21.43)	0.212	1.72 (0.67-4.36)		
Median no. days of antibiotherapy after onset of episode (other than the treatment for CDAD)	0 (0–0)	0 (0–0)	0.834	1.00 (0.92-1.10)		
Median no. antibiotics after onset of episode (other than the treatment for CDAD) [no. (IQR)]	0 (0–0)	0 (0–0)	0.746	1.06 (0.73-1.55)		
**Treatment for CDAD**						
Metronidazole [n (%)]	37 (22.02)	5 (11.9)	0.142	0.48 (0.14-1.35)		
Vancomycin [n (%)]	47 (27.98)	11 (26.19)	0.816	0.91 (0.39-2.08)		
Vancomycin + Metronidazole [n (%)]	75 (44.64)	24 (57.14)	0.146	1.65 (0.79-3.46)		
Rifaximin - α (alone or in combination) [n (%)]	7 (4.17)	2 (4.76)	1	1.15 (0.11-6.35)		

## Discussion

The high incidence identified in our study – 20.57/15.70 to 1,000 discharged patients in 2013/2014 or 17.73/14.04 to 10,000 patient-days - is explained by the endemicity in some hospitals in Timisoara, released in late 2012, and the fact that “Victor Babeş” Hospital is the only one in our area that provides treatment in all suspected or confirmed cases of this condition requiring hospitalization. The incidence was significantly higher in the age group over 60 years, but cases in young patients were also reported (lowest reported age was 19 years).

In Romania, the ”Matei Bals” Institute in Bucharest reported an increase in the number of infections with this germ from 1.67 cases/month in 2010 and 2 cases/month in 2011 to 60 cases/month in 2013, so that the average rate of 11.67 new cases/month identified by us between 2013–2014 is still lower [6,7].

In Hungary, the incidence of nosocomial CDAD was estimated at 1.2-2.8 to 10,000 patient-days during a 2011–2012 [8]. The EUCLID study conducted in 20 European countries, including 482 hospitals, shows a substantial increase in the incidence from 4.1 in 2008 to 6.8 cases/10,000 patient-days in 2013 [9]. In a Czech study published in 2014, CDAD was 1/10,000 patient-days, with a mortality rate of 22.4% [10].

Global fatality at 30 days varies between 9 and 38% (in a 2012 review) and between 8 and 31% (in a 2014 review) [11,12]. In our study, fatality at 30 days was 21.42% and recurrence was observed in 20% of patients.

The link between antibiotic use and hospital acquired CD infection has been supported by a number of epidemiological studies conducted since the 1990s. In hospital settings, the main risk factor is represented by the requirement for antibiotic treatments which, in the absence of careful clinical monitoring and epidemiologic surveillance, may become the source of ”serial” CDAD cases. The repeated courses of antibiotics played a significant part – excessive use of antibiotics is responsible for the destruction of the normal intestinal microbial flora, with the consecutive selection and development of resistant strains of pathogens. In our study group, 145 patients (69.05%) received antibiotics before the onset of enterocolitis, most of them received III-rd generation cephalosporins, fluoroquinolones, carbapenems and β–lactams associated with β-lactamase inhibitor.

Old age is identified as a risk factor for complications and death in many other articles [10,13-18]. In our study, it maintained its risk factor status in the multivariate analysis as well, but with values close to the limit. The Charlson comorbidity index includes various comorbidities and is generally used as an indicator to assess the risk of dying within 1 year. It is associated with the unfavourable outcome of the CDAD in an article from 2013, designed by the study group of *Clostridium difficile* in Barcelona, along with the age and continuation of the antibiotic treatment after the onset of enterocolitis [3]. In our study, the multivariate analysis does not retain the comorbidity index among the 30-day fatality risk factors, as we have seen in other studies [19,20]. This result may shift clinicians’ attention from age and comorbidity (which are little modifiable, if at all) to other extrinsic factors that can have a positive influence on the patient's evolution. The ATLAS score, predictive of poor outcome in our study (OR = 4.97, 95% CI = 2.12 to 11.66, p <0.001) included the following variables: age > 60 years, body temperature > 37.5°C, leukocytosis ≥ 16,000 cells/μl, hypoalbuminemia <3.5 g/dL and administration of systemic antibiotics (other than those for CDAD). Leukocytosis is incriminated not only in Alexey Markelov's studies [15,21] but also in those of Bhangu S. or Im G.Y. [17,22]. Hypoalbuminemia occurs as a variable associated with increased risk of mortality in several articles in recent years [10,17,21]. CDAD is traditionally associated with the consumption of clindamycin, III-rd generation cephalosporins and penicillins, and more recently with fluoroquinolones [23]. In a study conducted by Balihar Karela et al., high incidence was associated with consumption of penicillin but negatively correlated with the consumption of nitroimidazoles [10]. Continuing antibiotic administration after the onset of CDAD (other than for the treatment of enterocolitis) is incriminated in the study of Dolors Rodríguez-Pardo and his colleagues (2013) but also in the one from 2009 of the team led by Hu M.Y [3,24]. Our study identified, as a risk factor, the number of antibiotics administered after CDAD onset, other than the treatment for enterocolitis. (OR = 2.692, 95% CI = 1.01 to 7.17, p = 0.047).

We have not found any direct association between severe evolution and malignancy, but univariate analysis of variables retains chemotherapy on the list of significant risk factors.

In the literature, the increased risk of recurrence is associated with the age, leukocytosis over 15,000 cells/μl, continued treatment with proton pump inhibitors after the onset of CDAD or antibiotics concomitant with CDAD therapy, severe/fulminant clinical form and antitoxin A IgG <1.29 ELISA units [3,24].

The limitations of our study arise from the use of insufficiently sensitive diagnostic tests, resulting in a high percentage of false negative samples and, hence, the probability of missing the unconfirmed cases. By repeating the test at 48–72 h in patients with suggestive symptoms and epidemiological context, we tried to reduce the impact of the lower sensitive immunochromatographic methods as much as possible. The fatality rate could influence the analysis of recurrence risk factors.

## Conclusions

*Clostridium difficile* is a public health problem that has also emerged in Romania in the past three years. The emergence of this infection means an increase in the average duration of hospitalization and inpatient care costs, an increased number of deaths, especially among elderly patients, and compromising the effectiveness of medical interventions performed in hospitals where outbreaks of CDAD are evolving.

Our study identified the ATLAS score, age, and number of antibiotics after episode onset as predictors of an unfavourable outcome, while the number of days of hospitalization was associated with symptom recurrence.

## Abbreviations

CDAD: *Clostridium difficile*-associated disease
CD: *Clostridium difficile*
IQR: Range of quartile
OR: Odds ratio
95% CI: 95% confidence interval
